# Surface Characterization of Stainless Steel 316L Coated with Various Nanoparticle Types

**DOI:** 10.1155/2023/3997281

**Published:** 2023-01-25

**Authors:** Dhiaa J. Aldabagh, Thair L. Alzubaydi, Akram F. Alhuwaizi

**Affiliations:** ^1^Department of Orthodontics, College of Dentistry, University of Baghdad, Baghdad 00964, Iraq; ^2^Department of Prosthodontic Dental Technology, Al-Esraa University College, Baghdad 00964, Iraq

## Abstract

**Background:**

Material tribology has widely expanded in scope and depth and is extended from the mechanical field to the biomedical field. The present study aimed to characterize the nanocoating of highly pure (99.9%) niobium (Nb), tantalum (Ta), and vanadium (V) deposited on 316L stainless steel (SS) substrates which considered the most widely used alloys in the manufacturing of SS orthodontic components. To date, the coating of SS orthodontic archwires with Nb, Ta, and V using a plasma sputtering method has never been reported. Nanodeposition was performed using a DC plasma sputtering system with three different sputtering times (1, 2, and 3 hours).

**Results:**

Structural and elemental analyses were conducted on the deposited coatings using XRD, FESEM, and EDS showing a unique phase of coating metals over their substrates with obvious homogeneous even deposition. A highly significant positive correlation was found between sputtering time and thickness of the achieved coatings. AFM revealed a reduction in the surface roughness of 316L SS substrates sputtered with all coating materials, significantly seen in V coatings.

**Conclusions:**

Sputtering time and coating material play a significant role in terms of microstructure and topography of the achieved coatings being the best in the Ta group; moreover, surface roughness was significantly improved by V coatings. Likewise, it is found to be sputtering time independent for all used coatings.

## 1. Introduction

The study of friction, wear, and lubrication is included in the field of tribology, which is known as the “science and technology of interacting surfaces in relative motion.” Additionally, biotribology is typically defined as the study of tribological phenomena that occur in either the human body or in animals [1]. Biomedical materials are typically prepared to be suitable for use as medical devices or as part of their components and are intended to be in long-term contact with biological tissues. Studying the tribological aspect of these materials is important to increase their longevity by reducing wear and friction with biological tissues or other biomedical materials [2]. The term “medical device” refers to a broad category of healthcare items that play a significant role in the field of medical and health technology [3]. Stainless steel (SS), titanium (Ti), and cobalt-chromium-molybdenum (Co-Cr-Mo) alloy are the most commonly used materials in biomedical engineering because of their biocompatibility and mechanical qualities [4]. Metals and alloys have exceptional qualities and provide a wide range of options for fabricating orthodontic equipment, such as brackets, wires, bands, and ligatures. However, these materials have downsides that might cause issues for the orthodontist. In employing metallic alloys to prepare orthodontic equipment, some of the most prevalent drawbacks are poor friction control, allergic responses, and metal ion release. To address these flaws, researchers have developed various techniques, including coatings and surface treatments, to improve the suitability of these materials for orthodontic applications [5].

Orthodontic archwires could be manufactured using different materials, such as SS, Ti, Co-Cr-Mo [6], and esthetic materials [5]. Austenitic SS (316-316L) is the most common type of SS used to manufacture orthodontic brackets and archwires [7, 8]. During orthodontic treatment, friction is one of the most common deterrents to tooth alignment or retraction. This drawback can be overcome by applying high forces; however, the process may result in unwanted anchorage loss. Other options include changing the wire size and form, altering the bracket design, and coating the wire surfaces with various materials to overcome slide resistance. These coatings are applied to the bracket surface, SS, and nitinol (NiTi) wires [9]. Studies on understanding and managing friction during the use of orthodontic appliances have become popular. Frictional forces can work in conjunction with active tooth-moving forces or as a result of the tying mechanism's restriction. Understanding friction can help us choose a new appliance or enhance our knowledge of how to utilize an old appliance system [10]. During clinical tooth movement, friction depends on the material of orthodontic archwires and brackets [11], diameter in addition to cross-sections, surface roughness of archwires [12], bracket types [13], angulation between the wire and bracket [14], ligation types [15], environment (wet or dry) [16], and surface coatings [17]. More than 60% of the orthodontic force used to produce tooth movement is likely to be lost due to frictional forces [18]. Moreover, the proportion of applied force lost due to friction ranges from 12% to 70% [19].

For the synthesis of materials with improved tribological properties, the modified surface must have a suitable combination of qualities, such as adhesion, hardness, and strength, depending on the application. Understanding the benefits and shortcomings of the selected approach is necessary to accurately forecast the proper combination of desired surface microstructure and attributes [20]. Nanotechnology employs materials with dimensions no larger than 100 nm. With the advancement of the industrial, commercial, and agricultural sectors and pharmaceutical and medical sciences, individuals have significantly enhanced their lifestyles, and this technology has advanced quickly [21, 22]; however, their properties in nanoscales are greatly improved in comparison to their bulk material [23]. Moreover, surface modification can be used efficiently to alleviate potential risk of using inorganic biomaterials [24]. Coating is a unique way of altering the surface characteristics of a matrix, allowing it to adapt to a specific working environment and extending its service life [25]. This procedure can be defined as a thin coating produced or formed on the surface of a component made of another material that exhibits mechanical characteristics similar to those of the matrix and has high corrosion and wear resistance [26]. One of the well-known coating techniques is physical vapor deposition (PVD), in which materials are evaporated or sputtered to generate atoms, molecules, or ions, which are subsequently transferred to the substrate surface to form a thin film. This technique features high coating density, good adhesion, multicomponent layers, low substrate temperature, and efficient manipulation for a range of substrate and coating materials [27]. PVD can be divided into three types: evaporation, ion plating, and sputtering [28]. Sputtering involves the ejection of atoms or molecules by bombardment with high-energy ions, resulting in the deposition of a thin film of biomaterial. This approach enables good adhesion between the coating and the substrate, good control over the coating's characteristics, and enhancement of mechanical qualities, such as wear and corrosion resistance, biological activity, and biocompatibility [29]. Some crucial issues have been related to coating, and the most notable ones are delamination and coating deterioration. Nonetheless, research on appropriate materials and approaches to improve the characteristics of metallic biomaterials is still ongoing [30]. The substantial reduction in frictional resistance at the bracket-wire interface is one of the most significant benefits of coating archwires with nanoparticles [31].

To the authors' knowledge, no study has focused on the coating of SS archwires with Nb, Ta, or V by plasma sputtering. Therefore, the present research article aimed to assess the validity of the plasma sputtering system (including sputtering conditions and times) and the efficiency of the nanocoatings with Nb, Ta, and V coating metals deposited over 316 L SS substrates, the most widely used alloys in the manufacturing of SS orthodontic archwires.

## 2. Materials and Methods

### 2.1. Materials

Substrates of 316 L SS (15 × 15 mm and 2 mm thickness) were cut evenly in accordance with ASTM E3-95. Their chemical compositions were analyzed in accordance with ASTM E-1086 using spark atomic emission vacuum spectrometry, and the results are listed in Table 1.

All specimens were abraded successively using SiC grinding paper with different grits (80, 120, 230, 400, 600, 800, and 1000) to obtain a flat and scratch-free surface. Polishing was performed by diamond suspension (1, 3, 6, 9, and 15 *μ*m) until a smooth and mirrored surface with 150.2 nm surface roughness was obtained, followed by cleaning with ethanol of 99.8% for several minutes prior to sputtering. Tantalum (Ta), niobium (Nb), and vanadium (V) sheets of high purity (99.9%) (GOODFELLOW METALS Com., England) were selected as targets in the sputtering procedure to generate nanoparticles.

### 2.2. Nanocoating Procedure

Nanocoating was performed in a low-pressure gas discharge unit (plasma sputtering system) consisting of a vacuum chamber with two parallel electrodes and a cathode (on which a secured target of Nb, Ta, or V was fixed) that faces an anode disk (on which the substrates were placed). Both components were made of SS and had a distance of 50 mm between them, providing an electric field for the discharged argon gas. In this way, the sputtering beam will be sputtered evenly over the whole specimens. The plasma chamber was evacuated to a vacuum pressure of 10^−5^ mbar, argon gas was then introduced into the evacuated chamber, and the flow rate was adjusted until the pressure stabilized at the desired level (6 × 10^−2^ mbar). The voltage of DC power supply and discharge current were increased until plasma was generated at 25 watts of energy (7 mA × 3.6 kV). Each sputtering group cycle lasted 1, 2, or 3 hours. The DC power supply was switched off after the sputtering procedure, and the pressure was increased until it returned to normal. The specimens were then removed from the chamber, handled carefully, packed inside plastic containers, coded, and stored correctly to prevent damage and contamination.

### 2.3. Characterization of Nanocoated Surfaces

#### 2.3.1. Qualitative Analysis

*(1) Phase Detection*. The X-ray pattern of the crystalline substance can be regarded as a “fingerprint”; that is, each crystalline material has a unique diffraction pattern. XRD was performed using a D8 ADVANCE diffractometer (Bruker Com., Germany) that generates Cu K-*α*X-rays. Data were collected over a 2*θ*° angle ranging from 10° to 80° for a period of 787 seconds, *λ* = 1.5418 Å, with the scanning speed adjusted to 5°/min. Peak indexing was carried out based on the JCPDS (Joint Committee on Powder Diffraction Standards) using Match!3 software.

*(2) Topographical Morphology*. The shape, arrangement, and features of the nanoparticles were viewed using FESEM of MIRA 3 XMU (TESCAN, Brno, Czech Republic) under low vacuum conditions (combined with an EDS detector) with an accelerating voltage of 15.0 KV. Four magnification powers in the range of 25.0–150.0 KX were used, the view fields ranged from 1.38–8.30 *µ*m, and the scales ranged from 200–2000 nm.

#### 2.3.2. Quantitative Analysis

*(1) Thickness of Nanocoated Surfaces*. A cross-sectional analysis of the nanoparticle layers of the prepared nanocoated specimens (from each group) was performed using FESEM with an accelerating voltage 15.0 kV. Triple measurements at three different magnification powers ranging from 10.0–50.0 KX, a view field of 4.15–20.8 *µ*m, and a scale range of 1000–5000 nm were used to measure the thickness of the nanocoated surface layer.

*(2) Elemental Composition Percentage*. Elemental composition percentage was analyzed by energy-dispersiveX-ray microanalysis, in which the X-ray EDS spectra attached to FESEM were collected from each examined specimen under 15 kV accelerating voltage using an area analysis mode at 25 KX magnification, 8 × 8 *µ*m sampling window, and 100-second acquisition time. Quantitative analysis of the percentage of weight concentration was performed with IDFix-EDAX software by nonstandard analysis using ZAF correction methods.

*(3) Surface Roughness*. Surface roughness was analyzed using the AFM of the Brisk model (Ara Research Com., Iran) with a probe series of HQ-NSC15-ALBS, 8 nm radius of the typical uncoated tip made of silicone with an aluminum backside coating of the probe, a typical resonance frequency of 325 kHz (range of 265–410 kHz), and a typical spring force constant of 40 N/m (range of 20–80 N/m). Surface roughness parameters were measured using software as an integral part of AFM. The noncontact AFM mode (NC-AFM) was applied to analyze the surface roughness by utilizing the attractive interatomic force between the tip and a sample surface in three different areas, 2 × 2, 5 × 5, and 10 × 10 *µ*m, of each tested specimen from each sample group. The average surface roughness was then calculated. Moreover, the surface roughness of the untreated polished mirrored surface (10 × 10 *µ*m) of the 316L SS substrate specimens was analyzed for comparison with the nanocoated specimens.

## 3. Results

### 3.1. Qualitative Assessment

#### 3.1.1. Phase Detection

**Figure 1:**
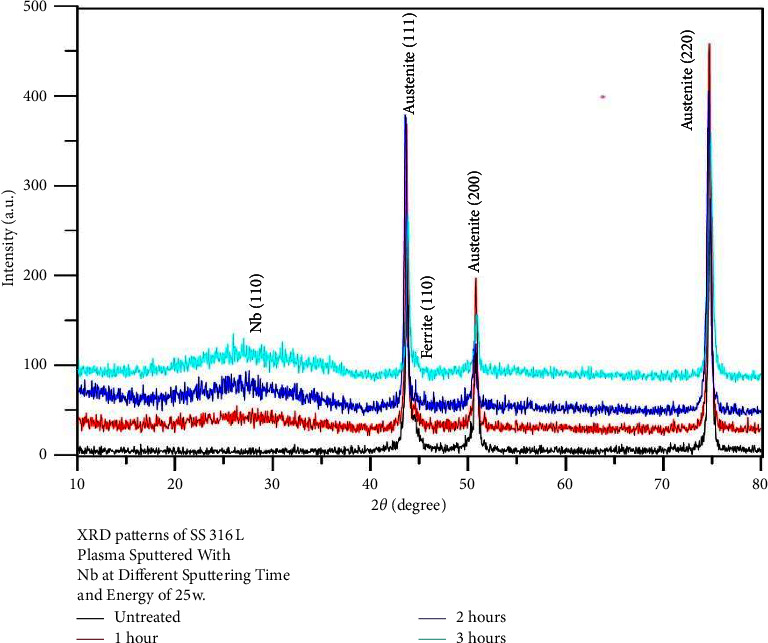
XRD patterns of 316L SS plasma sputtered with Nb at different sputtering times.

**Figure 2:**
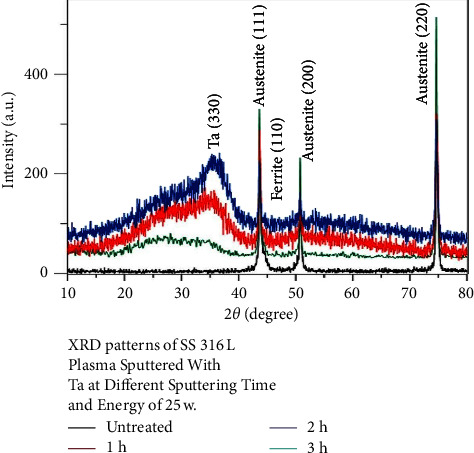
XRD patterns of 316L SS plasma sputtered with Ta at different sputtering times.

**Figure 3:**
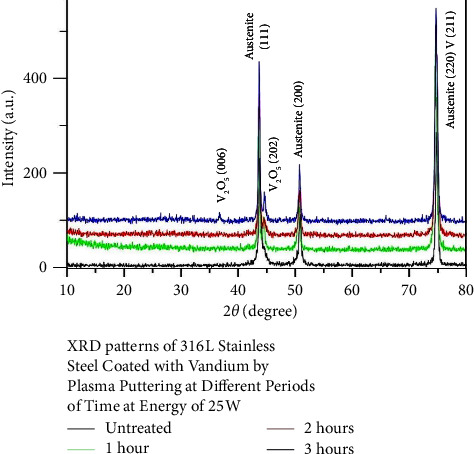
XRD patterns of 316L SS plasma sputtered with V at different sputtering times.

Figures 1–3 show the XRD patterns of all examined nanocoated specimens. The deposition of a coating layer of each target was confirmed by indexing the powder diffraction files and comparing them with the CPDS-ICDD files of Nb, Ta, and V, namely, #350789, #251280, and #221058, respectively, using Match!3 software.

#### 3.1.2. Topographical Analysis

**Figure 4:**
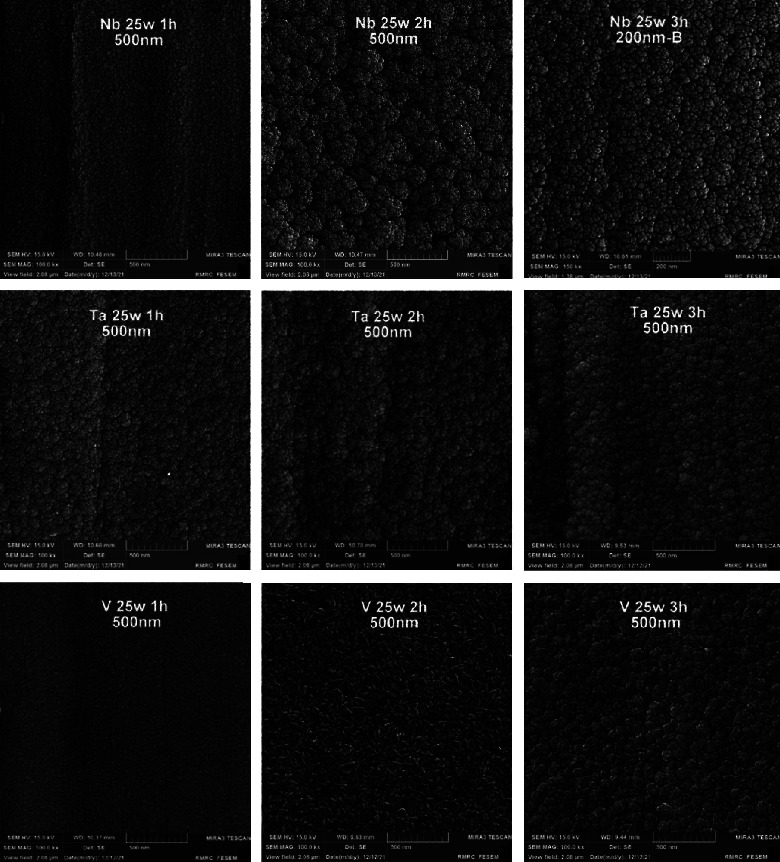
Morphology of the nanocoating particles of Nb, Ta, and V at different sputtering times.

Figure 4 presents the sputtered nanoparticles of different coating materials (Nb, Ta, and V) at different coating times (1, 2, and 3 hours) which were uniformly and densely distributed over their substrates. However, no gaps or voids were present among the nanoparticles of the coated layer. Moreover, the most prominent nanoshape seen was spherical, and the nanoparticles agglomerated in different patterns, mostly in nanoclusters. One exception was the V group, in which nanorods predominated and agglomerated in clusters.

### 3.2. Quantitative Assessment

#### 3.2.1. Thickness of Nanocoatings

Table 2 presents the estimated thickness of the nanocoated layer in different coating groups and subgroups. The lowest thickness was observed in the V 1-hour subgroup (190.6 nm), while the highest thickness was found in the Ta 3-hour subgroup (1544.5 nm). Moreover, the one–way ANOVA statistical test was conducted to compare the thickness of the nanocoating layers of the whole coated groups (Nb, Ta, and V) at different sputtering times, and there were significant differences among them. However, Tukey's post hoc tests in Table 3 show significant differences among the whole coated specimens at 1, 2, and 3 hours of sputtering time.

**Figure 5:**
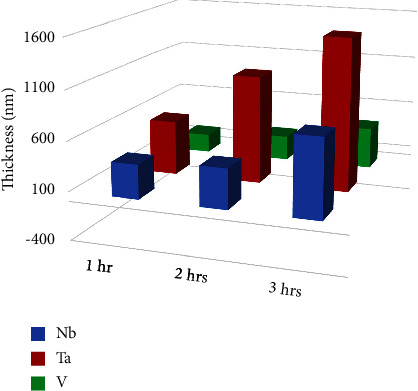
Coating thickness of Nb, Ta, and V coating materials at different sputtering times.

**Figure 6:**
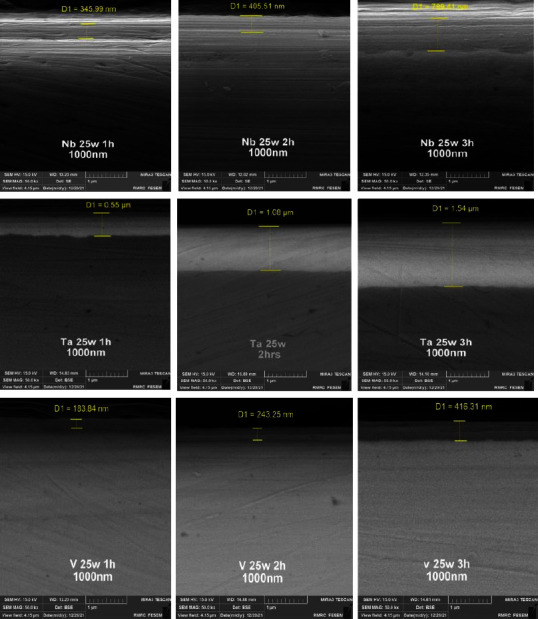
Thickness of Nb, Ta, and V nanocoating layers of plasma-sputtered 316L SS substrates (1^st^ readings at 1, 2, and 3 hours).

Figure 5 shows the average coating thickness of each group, and the highest was found in the Ta group which was reduced nearly to the half in the Nb group and further reduced nearly to the half in the V group.  Figure 6 shows examples of coated thickness readings at different sputtering times.

#### 3.2.2. Elemental Composition

**Figure 7:**
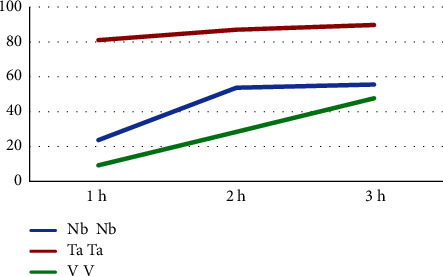
Elemental composition (wt.%) of coating Nb, Ta, and V at different sputtering times using the EDS test.

**Figure 8:**
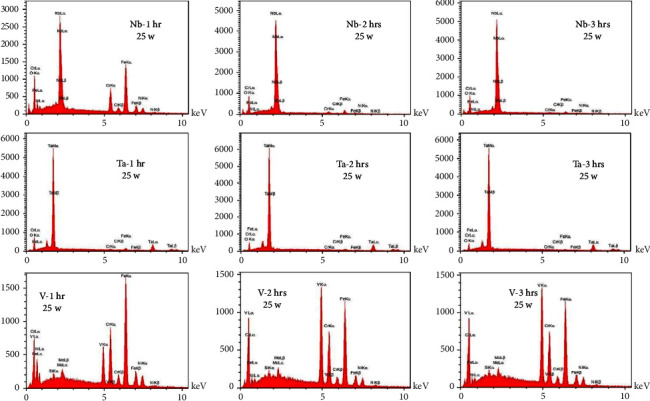
EDS of Nb, Ta, and V nanocoated specimens at different sputtering times.

According to Table 4, the highest wt. % of the coating material was found in the Ta 3-hour subgroup (90.1%), while the lowest was seen in the V -hour subgroup (9.56). Moreover, Figure 7 presents the deposition of coating elements which was the highest in the Ta group, followed by the Nb group, while the least was in the V group.  Figure 8 presents the results of the EDS test of the sputtered specimens of Nb, Ta, and V at different sputtering times.

#### 3.2.3. Surface Roughness

**Figure 9:**
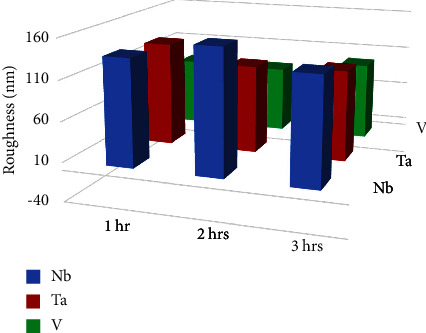
Comparison between sputtering time and surface roughness of Nb, Ta, and V coatings at 1, 2, and 3 hours of plasma sputtering.

**Figure 10:**
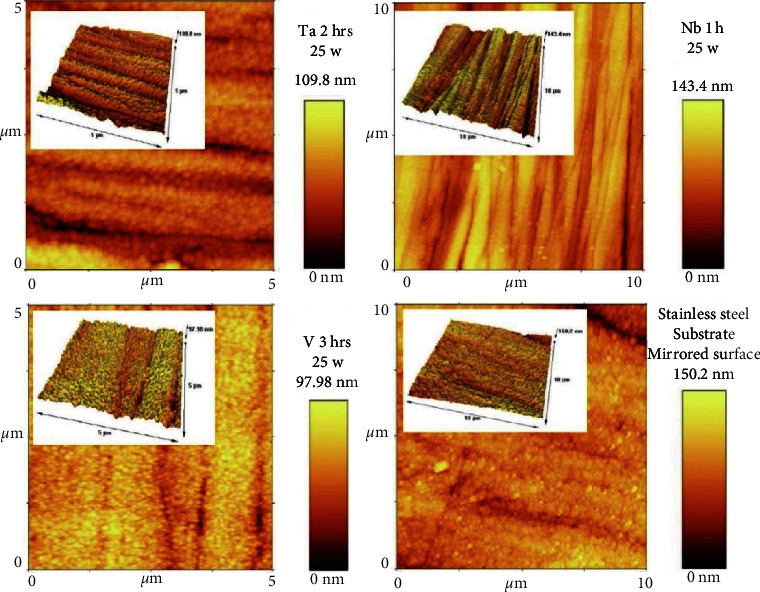
Comparison of 2D and 3D AFM topographical images of some specimen groups and untreated mirrored surfaces of 316L SS substrates.

Table 5 shows surface roughness for each coated specimen at different sputtering times (1, 2, and 3 hours). The least value was observed in the V2hr nanocoated specimens (83.4 nm). Generally, Figure 9 presents that Nb coating had the highest surface roughness followed by Ta coating, while the least surface roughness was seen in V coating, all being less than the roughness of the uncoated 316L SS substrate (157.8 nm).  Figure 10 shows the comparison of 2D and 3D AFM topographical images of some specimen groups and the untreated mirrored surface of 316L SS substrates.

Table 6 shows the comparison of surface roughness of the coated specimens at 3 different sputtering times (1, 2, and 3 hours) of different coating materials (Nb, Ta, and V) using the one-way ANOVA test. There were nonsignificant differences among each coated subgroup, while Table 7 shows that the surface roughness of the V group was the only significantly improved coating among the all coated groups in comparison to the uncoated 316L SS substrates using the independent sample *t*-test.

#### 3.2.4. Correlation between Sputtering Time and Coatings Thickness and Roughness

Table 8 presents highly positive significant correlations between sputtering time and thickness of coatings, while weak correlations are found between sputtering time and surface roughness of the achieved coatings.

## 4. Discussion

Friction during orthodontic treatment is one of the most vital issues in orthodontics, and many trials and efforts have been directed to gain this substantial goal, starting with the choice of material types having the least friction or the modification in biomechanics that led to the invention of the self-ligating bracket. Moreover, during any orthodontic treatment, the substantial goal is usually directed toward efficient teeth movement (acceptable rate and direction with the least hazards or risks of abnormal root and alveolar bone resorption). The most interesting effort that widens the scope of controlling friction is a surface treatment, namely, nanocoating of orthodontic appliance components, which is considered the most recent method to improve the tribology of these components. It is found that coated substrates reported friction reduction by up to 66% when compared to uncoated ones [32]. Many factors may govern this approach. The hardness of coatings is considered a substantial factor that governs the friction of opposing surfaces; therefore, it is assumed that the selection of harder material may play an important role in friction reduction [33]. Therefore, in the present research article, the Mohs scale was used primarily to select suitable harder coating metals (Ta = 6.5 Mohs, V = 7 Mohs, and Nb = 6 Mohs) [34]. The Mohs scale is considered the oldest reliable hardness measurement method for more than a century which is still valid, devised by Friedrich Mohs in 1812 [35], who proposed ten minerals in increasing order of scratch hardness (softest:1. talc, 2. gypsum, 3. calcite, 4. fluorite, 5. apatite, 6. orthoclase, 7. quartz, 8. topaz, 9. corundum, and 10. diamond), and is used as an aide to classify minerals and other materials based on their hardness. At first glance, it would seem that such a scale might be completely arbitrary and devoid of any fundamental physical significance, but he reported that it is possible to construct a scratch hardness scale where each standard has indentation hardness that is at least 1.2 times greater than the standard before it [36, 37]. Moreover, the biocompatibility of the used coatings is considered the keystone in the selection of any biomedical coating material to be used; however, Nb, Ta, and V have been used in the medical field for more than 10 years [38–40]. Therefore, Nb, Ta, and V were selected accordingly.

Many surface treatment methods can be used to coat a bulk material; however, sputtering, which is a type of PVD, might be preferred to others due to the following reasons:

1. It is a physical procedure; therefore, the structural properties of the substrates will not be affected, in addition to their simplicity and versatility [41].
2. Regardless of the type, sputtering reduces delamination and provides good adhesion to their substrates with higher coating density [27, 29].
3. Broad spectrum types of metals and/or metal alloys can be used as a source for nanoparticle production to fabricate suitable thin film coating layers [27, 42].
4. Coating's properties can be controlled by varying the sputtering time and/or conditions [42, 43]. The most important orthodontic components requiring surface treatment are the orthodontic archwires and brackets since they are lying in the friction zone [9]. Friction usually occurs in any type of tooth movement whatever the direction of the moved tooth and the used orthodontic archwire; however, the highest friction may be faced during space opening and/or closure (translation orthodontic tooth movement) [10] in which the most probable used archwire is made of stainless steel. Hence, the present research article focused on 316L SS substrates which are considered the most widely used SS alloys in the manufacturing of orthodontic brackets and archwires [7, 8, 44].

Surface properties of alloys and metals can be improved greatly by the deposition of coatings, specifically hard coatings [45]. The XRD test of all nanocoating specimens indicated the presence of a specific fingerprint of coated elements (Nb, Ta, and V) over their substrates in addition to Fe (the main constituent of the SS substrate). Moreover, FESEM of all tested specimens including surface topography and cross-sectional analysis showed the deposition of even, dense, and homogeneous coating layers with the absence of gaps or voids, since most of the failures observed in any material are primarily due to the presence of surface voids or gaps. These parameters are considered the most substantial properties that affect the mechanical and electrochemical features of nanocoatings and govern their durability and proper functioning. The main drawback of coatings is the probability of delamination [30], usually indicated by the presence of surface gaps or voids among aggregated nanoparticles (regardless of their types) that were not detected in the topographical analysis of all tested specimens (Figure 4), as well as the presence of internal gaps, voids, or signs of delamination between coating layers and their substrates that were completely absent in the cross-sectional analyses of all tested specimens (Figure 6). Therefore, phase detection and topographical analysis of all coated specimens implied the efficiency of the used sputtering materials and conditions, in agreement with the findings of Mattox [27].

Varying elemental compositions (wt.%) of the target metal elements were deposited on their corresponding substrates and detected using the EDS test, being the highest in the Ta group, reduced nearly to half in the Nb group, and the least found in the V group. Since the X-ray depth in the EDS test generally does not exceed 2 *µ*m [46, 47], the maximum coating thickness achieved in all tested groups was less than 2 *µ*m. Therefore, the elemental composition (wt.%) of the target metal elements (Nb, Ta, and V) in all analyzed specimens increased with the sputtering time (Table 9). By contrast, a reduction was observed in Fe (which forms the bulk constituent of 316L SS alloy), coincided with the finding of Cao and Zhou [48], indicating efficient deposition of coatings elements over the surface of their substrates.

The highest coating thickness was observed in the Ta group, which was reduced to nearly 50% in the Nb group and further reduced to nearly 50% in the V group (Table 2 and Figure 5) at varying sputtering times (1, 2, and 3 hours), which might be attributed to the following conclusions:

1. A highly positive and significant correlation was found between sputtering times and coating thickness for each coated group (Nb, Ta, and V), which depends on the rate of nanoparticle deposition since the sputtering procedure depends on the bombardment of targets (Nb, Ta, and V in the present study) by energetic particles from an inert gas (argon), leading to ejection (sputtering) of nanoparticles from the target to be deposited on the substrates (316L SS); hence, for each group, targets were used separately. It is found that the increment in nanomaterials will be increased if the sputtering time is extended from 1-2 or 2-3 hours, especially when the other sputtering conditions like sputtering energy, target-substrate distance, and sputtering pressure are identical, in agreement with the finding of Sproul [42] who reported that thickness of the thin coat can be increased with increasing sputtering time.
2. Surface binding energy (SBE) of the used coating metals (Nb, Ta, and V), which is considered the most important parameter that controls the ion sputtering process and the final composition of the sputtered coating surface, is defined as the required energy to be applied on the target to eject an atom from the top surface layer during the ion sputtering procedure in vacuum [49]; interestingly, it is found that Ta metal has the least SBE (21.8 eV) among the used targets, while V metal has the highest SBE (512.3 eV). Moreover, Nb metal has medium SBE (202.4 eV) [50], which might explain logically the real reason of elemental compositional (wt. %) and thickness variations among the used targets, being the highest in the Ta group and the least in the V group, while the Nb group had the medium level at an identical sputtering time, coincided with the finding of Kudriavtsev et al. [49] and Dowling et al. [51]. This revealed the importance of selecting the best coating material and sputtering time to be analyzed subsequently for their effects on the tribological features (microhardness, friction, and wear) of the achieved coatings which considered the final substantial goal of coating 316L SS substrates that considered the most common metal alloys used to manufacture metal orthodontic appliance components.

Regarding surface roughness assessment of the achieved coatings, AFM is still considered the most suitable tool for evaluating surface topography because it can provide 3D images [52] and has many advantages, such as high-resolution 3D assessment, simplicity of testing surface topography (the analyzed specimen does not require any laboratory preparation, such as metallization), and the ability to quantitatively assess surfaces. Meanwhile, its main drawback is the limited area that can be analyzed, hence failing to assess some unselected regions with roughness [53]. Comparison among coating subgroups regarding sputtering times presented nonsignificant differences among them, certified by the presence of a weak correlation between sputtering times and surface roughness of all coated specimens, illustrating that surface roughness tends to be time independent in the present research study. This finding was in line with the finding of Vázquez et al. [54] and disagreed with the finding of Howlader et al. [55], who reported that surface roughness is usually increased with increased sputtering time, and with the finding of Paramanik et al. [56], who reported that elongation of sputtering time may lead to the reduction of surface roughness. Moreover, Table 8 shows an improvement in surface roughness generally for all the coating subgroups in comparison to 316L SS substrate roughness; however, V coating had the only remarkable significant roughness improvement using an independent *t*-test for the comparison with its substrate, which might be attributed to the homogeneity of nanoparticle arrangement of V that affects surface topography, leading to a smoother surface in comparison to Ta and Nb (Figure 4), in line with the finding of Beltrami et al. [57].

The main goal of the present research article is the enhancement of the tribology of SS substrates, which are the most commonly used alloys to manufacture orthodontic archwires to reduce friction with another part of the orthodontic appliance, namely, orthodontic brackets, through nanocoating by various metal (Nb, Ta, and V), hence achieving the goal of a prosperous orthodontic treatment to minimize orthodontic treatment time and unwanted risk sequelae. The argument of smoother materials is usually associated with a low coefficient of friction; nevertheless, it is not easy to understand this relationship. At their extremes, materials with high and low roughness can have large coefficients of friction. High coefficients of friction of highly polished surfaces are widely known. Therefore, intermediate roughness is the only one that has a good link with the coefficient of friction [8]. Therefore, the coefficient of friction of all the tested nanocoated groups must be analyzed to assess the effect of coatings on tribological properties (microhardness and wear) using a well-prepared scientific approach. This parameter will be addressed in the future part of this research study.

Research on the nanocoating of SS orthodontic archwires (mostly made of 316L SS) with Nb, Ta, and V using plasma sputtering and their subsequent effect on the friction between SS archwires and orthodontic brackets is scarce, hence underlining the novelty of the present research article approach.

## 5. Conclusion

Plasma sputtering for 316L SS substrates (the most widely used alloys for manufacturing SS orthodontic archwires) was performed by Nb, Ta, and V at different sputtering times (1, 2, and 3 hours), and surface characterization analyses revealed the following:

1. The deposited coated layer by all coating materials was even, homogeneous, and free from sign of delamination.
2. Elemental compositions and thickness of all coatings were time-dependent, increasing with sputtering time elongation, being the best in the Ta group.
3. Surface roughness of all achieved coatings improved generally in comparison to that of noncoated SS substrates, being significantly obvious in the V group; moreover, variation in the sputtering time had nonsignificant effects on the surface roughness of the coatings.

Selecting the proper type of coating material and suitable sputtering time plays a substantial role in the improvement of tribology (friction and wear) of the substrate (316L SS) which is the most widely used alloy for fabrication of SS orthodontic archwires, hence expecting to reduce friction between orthodontic archwires and other parts of orthodontic appliances, leading to optimal orthodontic teeth movement and reduction in time of treatment time and unwanted sequalae like teeth or alveolar bone resorption.

## Figures and Tables

**Table 1:** Elemental composition and weight% of tested SS substrates.

Elements	Cr	Ni	Fe	Mo	Mn	Co	Al	Ti	C
Wt.%	16.6	11.4	66.8	1.98	1.61	0.30	0.006	0.03	0.001

**Table 2:** Comparisons of nanocoating thickness at different sputtering times (1, 2, and 3 hours) and for different coating materials (Nb, Ta, and V) using the one-way ANOVA test.

	Sputtering time	1^st^ reading	2^nd^ reading	3^rd^ reading	Mean (nm)	SD±	ANOVA
Nb	1 hr	345.99	361.2	350.5	352.6	7.81	*F* = 1828.101 *P*=0.001
	2 hr	405.51	400.4	420.6	408.8	10.50	
	3 hr	789.41	795.5	810.6	798.5	10.91	

Ta	1 hr	550	570.4	542.8	554.4	14.32	*F* = 2245.684 *P*=0.001
	2 hr	1080	1086	1113.2	1093.1	17.79	
	3 hr	1540	1525.5	1568	**1544.5** ^∗^	21.60	

V	1 hr	183.84	190.8	197.3	**190.6** ^∗∗^	6.73	*F* = 580.255 *P*=0.001
	2 hr	243.25	260.5	250.1	251.3	8.67	
	3 hr	416.31	410.5	429.6	418.8	9.79	

ANOVA	1 hr	*F* = 960.192, *P*=0.001					
	2 hr	*F* = 3613.71, *P*=0.001					
	3 hr	*F* = 4330.497, *P*=0.001					

The mean difference is significant at the 0.05 level. ^∗^indicates the highest achieved coating thickness, while ^∗∗^indicates the lowest achieved coating thickness.

**Table 3:** Multiple comparisons of nanocoating thickness at different sputtering times (1, 2, and 3 hours) and for different coating materials (Nb, Ta, and V) using Tukey's post hoc tests.

Dependent variable	Independent variable		Mean difference	Sig
Nb	1 hr	2 hr	56.273^*∗*^	0.001
		3 hr	445.940^*∗*^	0.000
	2 hr	3 hr	389.666^*∗*^	0.000

Ta	1 hr	2 hr	538.666^*∗*^	0.000
		3 hr	990.100^*∗*^	0.000
	2 hr	3 hr	451.433^*∗*^	0.000

V	1 hr	2 hr	60.636^*∗*^	0.000
		3 hr	228.156^*∗*^	0.000
	2	3 hr	167.520^*∗*^	0.000

1 hr	Nb	Ta	201.836^*∗*^	0.000
		V	161.916^*∗*^	0.000
	Ta	V	363.753^*∗*^	0.000

2 hr	Nb	Ta	684.230^*∗*^	0.000
		V	157.553^*∗*^	0.000
	Ta	V	841.783^*∗*^	0.000

3 hr	Nb	Ta	745.996^*∗*^	0.000
		V	379.700^*∗*^	0.000
	Ta	V	1125.696^*∗*^	0.000

^*∗*^indicates that the mean difference is significant at the 0.05 level.

**Table 4:** Elemental composition (wt.%) of coated Nb, Ta, and V at different sputtering times using EDS.

Coated specimen	Elements	1 hr	2 hr	3 hr
Nb	Nb (wt.%)	24.06	54.11	55.95
Ta	Ta (wt.%)	81.46	87.41	90.1
V	V (wt.%)	9.56	28.77	48.07

**Table 5:** Surface roughness of coated (Nb, Ta, and V) specimens and untreated 316L SS substrates at 3 different sputtering times (1, 2, and 3 hours).

	Sputtering times	1st reading	2nd reading	3rd reading	Mean nm	SD±
Nb	1 hr	99.9	161.5	143.4	134.9	31.66
	2 hr	111.4	126.3	230.6	156.1	64.93
	3 hr	109.5	149.6	140.7	133.3	21.06

Ta	1 hr	93.7	80.6	217.5	130.6	75.54
	2 hr	46.4	109.8	173.8	110	63.70
	3 hr	59	127.8	152	112.9	48.23

V	1 hr	83.8	36.1	136.1	85.3	50.01
	2 hr	73.7	99.9	76.6	83.4	14.34
	3 hr	106.4	98	86.1	96.8	10.20

SS		154.8	168.5	150.2	157.8	9.52

**Table 6:** Comparison of surface roughness of all coated specimens at 1, 2, and 3 hours using the one-way ANOVA test.

	*F*	Sig
Nb	0.257	0.781
Ta	0.092	0.913
V	0.169	0.849

The mean difference is significant at the 0.05 level.

**Table 7:** Independent *t*-test between each coated group and uncoated 316L SS substrates.

	*N*	Mean (nm)	Std. deviation	*t*	*p* value
Nb	9	141.4	39.21	−0.696	0.502
SS	3	157.8	9.52		

Ta	9	117.8	55.82	−1.197	0.259
SS	3	157.8	9.51		

V	9	88.5	27.25	−4.202	**0.002**
SS	3	157.8	9.51		

The mean difference is significant at the 0.05 level. The bold value indicates a significant statistical difference present between surface roughness of untreated 316L SS and coated substrates by V nanoparticles.

**Table 8:** Sputtering time correlations with thickness and roughness of coatings.

Variables		Thickness			Roughness		
Coatings		Nb	Ta	V	Nb	Ta	V
Time	Pearson correlation	**0.917** ^*∗∗*^	**0.998** ^*∗∗*^	**0.963** ^*∗∗*^	−0.018	−0.137	0.183
	Sig. (2-tailed)	0.000	0.000	0.000	0.963	0.725	0.638
	*N*	9	9	9	9	9	9

^*∗∗*^indicates that the correlation is significant at the 0.01 level (2-tailed). The bold values indicate the presence of high positive correlations between coating thickness and sputtering times.

**Table 9:** Weight % of nanocoated elements corresponding to Fe present on the 316L SS substrate surface at different sputtering times using the EDS test.

Coated specimen	Elements	1 hr	2 hr	3 hr
Nb	Nb (wt.%)	24.06	54.11	55.95
	Fe (wt.%)	37.41	4.95	2.42

Ta	Ta (wt.%)	81.46	87.41	90.1
	Fe (wt.%)	2.63	1.09	0.59

V	V (wt.%)	9.56	28.77	48.07
	Fe (wt.%)	60.78	47	34.53

## Data Availability

The data used to support the findings of this study are available from the corresponding author upon request.
